# The Use of a Natural Polysaccharide Extracted from the Prickly Pear Cactus (*Opuntia ficus indica*) as an Additive for Textile Dyeing

**DOI:** 10.3390/polym16142086

**Published:** 2024-07-22

**Authors:** Lucia Emanuele, Mateo Miguel Kodrič Kesovia, Tanja Dujaković, Simone Campanelli

**Affiliations:** 1 Department of Art and Restoration, University of Dubrovnik, 20000 Dubrovnik, Croatia; lucia.emanuele@unidu.hr (L.E.); tanja.dujakovic@unidu.hr (T.D.); 2 School of Science Technology, Section of Chemistry, University of Camerino, 62032 Camerino, Italy; simone.campanelli@studenti.unicam.it

**Keywords:** polysaccharide, *Opuntia ficus indica*, dyes, natural additives, silk, colorimetric analysis

## Abstract

The art of dyeing fabrics is one of the oldest human activities. In order to improve the fastness properties of dyeing products, various additives are added to optimize the uniformity of fibers and surfaces and improve dye distribution. Unfortunately, these additives can be harmful and very often are not biodegradable. This article reports on the possibility of using a natural additive for dyeing textiles: a polysaccharide extracted from the prickly pear cactus (*Opuntia ficus indica*). One type of fabric was tested, silk, with different colors. Several samples were prepared and dyed for each color, adding the same additives but also a commercial chemical aid for one of them and the mucilage of Opuntia for another. The fastness of the applied dyes was evaluated by washing at different temperatures with a common liquid detergent. All samples were analyzed before and after washing with a colorimeter to evaluate the color changes. The results of the analyses reported and compared indicate the potential of prickly pear mucilage as an additive for dyeing silk, which is easily accessible, safe, and sustainable compared to other commonly used additives.

## 1. Introduction

The art of dyeing fabrics is one of the oldest human activities. The oldest civilizations obviously only used natural dyes, but starting from the 19th century, the synthetic dye industry established itself, although many artificial textile dyes are dangerous for the environment as well as for human health [1]. Additionally, in order to improve the fastness properties of dyeing products, various additives are added. Without additives, the water-soluble part of the dye molecule, which carries a negative charge, tends to repel the fibers, which become negatively charged in water at the surface. The use of additives can facilitate the dyeing process in various ways. Firstly, they increase the attraction between the dye and the fiber. They also have a balancing effect and ensure that the dyeing is more even. And finally, they promote the anchoring of the dye molecules in the fiber by reducing their desorption rate [2].

Unfortunately, these additives can be harmful and very often not biodegradable. It is known that wastewater from the textile industry contains harmful dyes as well as various pigments, heavy metals, sulfates, oils, surfactants, and chlorides [3].

Considering what has been said, it becomes clear why the textile dyeing industry is considered one of the main contributors to environmental pollution [4]. It is therefore not surprising that in recent years, research has become increasingly interested in new environmentally friendly and green materials, such as chitosan, nettle extract, and shellac in an ethanol solution, that can be used as additives [5,6] to reduce environmental impact.

We recently reported on the use of a natural polysaccharide extracted from the prickly pear cactus (*Opuntia ficus indica*) as a color-fixing agent on watercolor paper. The gel extracted from the cladodes has been shown to act as a color fixative, reducing color loss on the paper treated with it. In view of this, we have decided to test the same polysaccharide as an additive for textile dyes [7].

**Figure 1:**
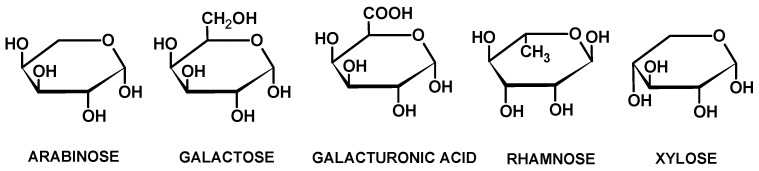
Molecular structures of the main sugars of the mucilage of *Opuntia ficus indica*.

*Opuntia ficus indica* is an ornamental plant that is cultivated in many countries around the Mediterranean. These plants are also used in the food and cosmetics industry due to their nutritional, medicinal, and cosmetic properties. For these reasons, the plants must be pruned regularly to remove the cladodes, which are then disposed of as waste. The chemical composition of mucilage of *Opuntia ficus indica* has been largely studied. It seems composed of 55 sugar residues, including arabinose, rhamnose, galactose, xylose, and galacturonic acid [8] (Figure 1).

In addition to the characteristic bands of polysaccharides, the FTIR spectrum of mucilage also shows contamination by proteins. This particular chemical composition indicates that mucilage can be used as a leveling agent in textile dyeing instead of conventional chemical agents.

For this reason, this paper presents the test procedure for this natural polysaccharide as an additive in silk dyeing processes. The whole study consists of three different test cycles. First, two sets of three silk samples were tested, dyed with two different dyes, keeping the same liquor ratio but using different additives: a commercial one and the mucilage of *Opuntia ficus indica* (hereafter referred to as “the first test”). Subsequently, two sets of two samples were tested with the same additive (only the mucilage), varying either the concentration or the liquor ratio (hereafter referred to as “the second test”). In the last test cycle (hereafter referred to as “the “third test”), we repeated the analyses with different additives and dyes and selected the highest liquor ratio for all samples.

The second part of the paper reports on the wash fastness analysis and comments on the differences.

It should be emphasized that the polysaccharide chosen for these analyses has been extensively studied and that its sustainability and biodegradability have been repeatedly confirmed, as well as the economic advantage of its use due to its low cost and availability [9,10,11,12].

## 2. Materials and Methods

### 2.1. Fabrics

**Figure 2:**
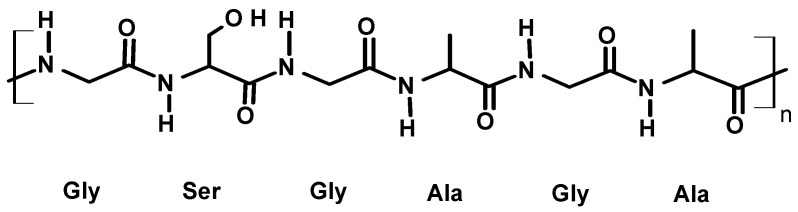
Molecular structure of silk fibroin.

The non-dyed textile used for the experiment was a protein fiber (Figure 2), pure silk pongee, 124.716 gms.

For the first test, two sets of three samples (8 × 20 cm^2^) were made, weighed, and labeled, as reported in Table 1.

For the second test, two samples (8 × 20 cm^2^) were prepared, weighed, and labeled, as reported in Table 2.

After the first two tests, a decision was made to substitute LANASET^®^ (Archroma, Huntsman Textile Effects Corporation, Hardstrasse 1, 4133 Pratteln Basel-Land, Switzerland) Bordeaux B dye with LANASET^®^ Red 2GA-01. This was decided in order to observe the color changes also on lighter shades. Another reason was that the official LANASET^®^ manual of dyes states that LANASET^®^ Red 2GA-01 dye has a slightly weaker fastness and resistance when rubbed and washed under different temperatures. Wet fastness on silk is generally lower than on wool. Such properties will elevate the threshold for testing the effectiveness of the natural additive [13].

For the third test, two sets of three samples (5 × 10 cm^2^) were prepared, weighed, and labeled, as reported in Table 3.

### 2.2. Colorants

The LANASET^®^ range consists of modified, strong 1:2 metal complex, acid and reactive dyes with high wet and light fastness, which are bound to the fiber via covalent bonds. They are ideal for numerous fiber types, including wool, polyester, polyamide, silk, acrylic, and cellulose [13].

Three different dyes were used to color the samples: LANASET^®^ Bordeaux B, LANASET^®^ Red 2GA-01, and LANASET^®^ BLUE 2RA.

### 2.3. Additives

**Figure 3:**
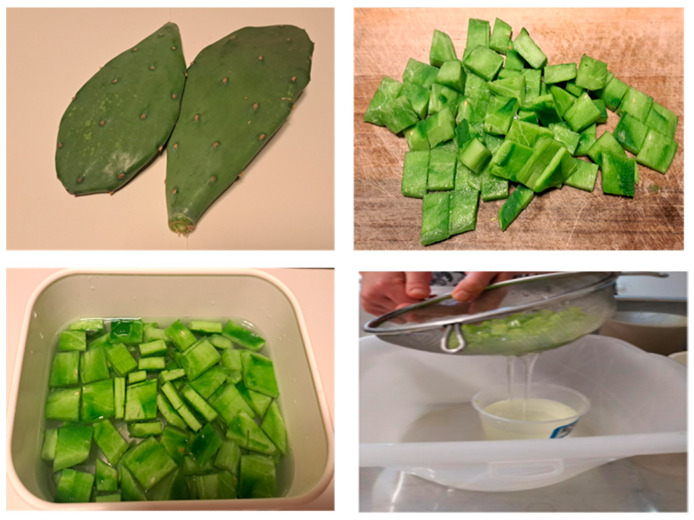
MA extraction method.

The natural additives tested on both fabrics are polysaccharides extracted in the form of mucilage from the prickly pear cactus. The cladodes used were collected from a native plant in the Dubrovnik region of Croatia. The mucilage was extracted using a variation of the maceration method (MA), which is one of the many methods described in the literature [14,15]. The cladodes are peeled and cut into small pieces in the order of 1 cm^3^. The cut pulp (50 g) is immersed in distilled water (250 g), and then the mixture is left to stand in the dark for 24 h. After filtration, a transparent and not very dense mucilage is obtained (Figure 3).

For all samples, further additives were added: Acetic acid, sodium sulfate, and sodium acetate for all three series of samples. Albegal SET^®^ (Archroma, Huntsman Textile Effects Corporation, Hardstrasse 1, 4133 Pratteln Basel-Land, Switzerland) was only added to one series, and cactus mucilage was added to another one. Albegal SET^®^ is a leveling agent used together with LANASET^®^ dyes to optimize the leveling of fibers and surfaces and to promote excellent dye distribution. It is important to mention that Albegal SET^®^ must be handled with personal protective equipment such as gloves and goggles and that it is also classified as harmful to aquatic organisms [16].

#### Chemical–Physical Characterization of the Mucilage

The substance extracted by the MA method has been characterized using FT-IR Alpha-II spectrometer by Bruker (Bruker corporation, Billerica, 40 Manning Rd, United States); the measurement range was 400–4000 cm^−1^ and the resolution was 4 cm^−1^ with 24 scans per sample.

**Figure 4:**
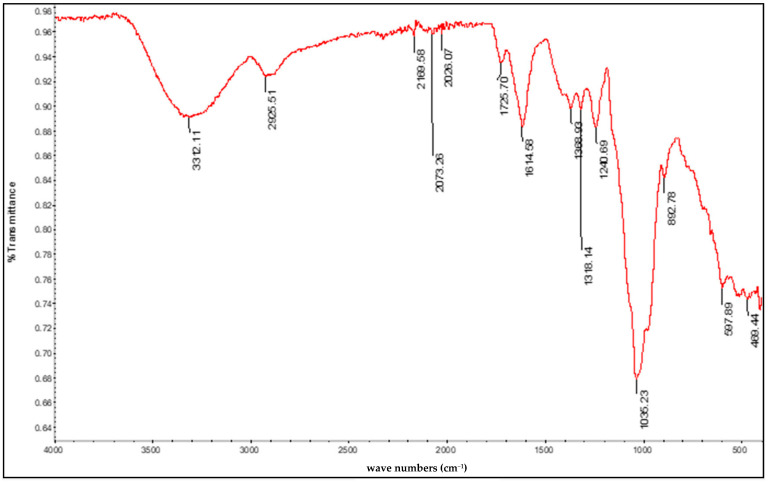
FTIR spectrum of the substance extracted from *Opuntia ficus indica* blades.

The recorded FTIR spectrum shows the characteristic bands of the mucilage of cactus cladodes (Figure 4) [17,18]. The stretching band around 3312 cm^−1^ and the bending band around 1425 cm^−1^ are both typical of the hydroxyl groups (-OH). The stretching band at 2925 cm^−1^ and the bending band with a peak at 1614 cm^−1^ are characteristic of the alkyl groups (-CH, -CH_2_, and -CH_3_). The bands observed at 1369 cm^−1^ and 1240 cm^−1^ are due to the characteristic vibrational modes of the pyranose ring. The absorption peak at 1035 cm^−1^ characterizes the single bond -C-O of the alcohols and the corresponding ether bridges in the main chain of the polysaccharide. In addition, the weak band at 1725 cm^−1^, which indicates a carbonyl group (-C=O), probably an amide group, suggests possible contamination by protein materials during extraction.

### 2.4. Dyeing Process

The samples were dyed according to a known multi-step procedure [2]: preparation for dyeing, formulas for dyeing, and dye cycles.

#### 2.4.1. Preparation for Dyeing

The preparation of the samples consists of a pre-wash aimed at removing any impurities on the tissue that could interfere with the binding of the dye to the tissue.

All the samples were washed in boiling water (ca. 800 mL) with a little laundry detergent (ca. 8 mL) for 20 min and then rinsed under running water.

#### 2.4.2. Formulas for Dyeing

Generally, for the silk samples, the volume of dye and additives are calculated using the following formulas:
$\mathrm{dye}\;\mathrm{volume}\;(\mathrm{ml})=\frac{W\times P_{d}}{C}$W=weight of fabric $P_{d}=\mathrm{shade}\;\mathrm{dye}\;\mathrm{percentage}$ C=% dye concentration$\mathrm{additive}\;\mathrm{volume}\;(\mathrm{ml})=\frac{W\times P_{a}}{C_{a}}$W=weight of fabric $P_{a}=\mathrm{additive}\;\mathrm{percentage}$ $C_{a}=\%\mathrm{additive}\;\mathrm{concentration}$$\mathrm{additive}\;\mathrm{volume}\;\mathrm{sodium}\;\mathrm{acetate}\;(\mathrm{ml})=\frac{\mathrm{TL}\times P_{a}}{10\times C_{a}}$TL=total liquor $P_{a}=\mathrm{additive}\;\mathrm{percentage}$ $C_{a}=\%\;\mathrm{additive}\;\mathrm{concentration}$

The recommended liquid ratio (LR) for silk is 1:40, which is the ratio used for the first test. For the second test, it was changed to 1:60, and for the last test, even to 1:100. The reason for this is to have the fabric samples fully submerged within the dye bath, i.e., unfolded and untwisted during the dyeing process, achieving thus much more homogeneous results.

The dye concentration (C) for all LANASET^®^ reactive dyes was 0.2%, while the shade dyes percent (P_d_) was 3% o.w.f.

The additive concentration (CA) was 10% for all additives, while the additive percentage (P_a_ for acetic acid, Albegal SET^®^, and sodium acetate) was 1% o.w.f. and 5% o.w.f. for sodium sulfate.

The samples obtained are still labeled as they were at the beginning, but now they have a new meaning (Table 4 and Table 5) based on the type of additives added and on the liquor ratio.

#### 2.4.3. Dye Cycles

The dyeing cycles consist of time intervals with different temperatures to control the dyeing process. Normally, the fibers dye better at high temperatures, but these could damage the fibers themselves, so it is necessary to control the temperature cycle and gradually increase and decrease the temperature. The temperatures and the time intervals are different for every kind of fabric.

##### Dye Cycle for Silk Samples

In the first phase, the samples are immersed in water at a temperature of 50 °C. At this temperature, the additives are added, and the pH value is measured. For all depths of shade, LANASET^®^ dyes are used at a pH value of 4.5, which corresponds to the isoelectric range of silk and leaves the fiber in an excellent physical condition. Dyeing at pH 4.5 and using higher amounts of salt can improve the depth of dye on silk [13].

**Figure 5:**
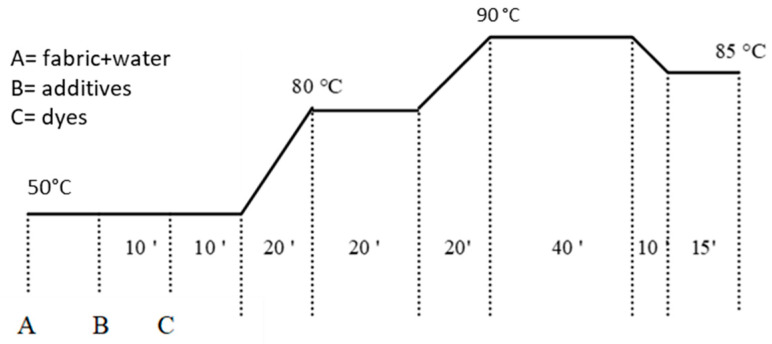
The diagram of time and temperatures for the dyeing cycles for silk samples.

This value is guaranteed by the use of acetic acid and sodium acetate, which together buffer the dye bath so that the pH remains constant throughout the entire cycle. Ten minutes later, the dyes are added. The temperature is then gradually increased to 80 °C, kept at this temperature for 20 min, and then gradually increased until a temperature close to boiling point (90 °C) is reached, which is then kept constant for about 40 min and then gradually lowered to 85 °C and kept constant for 15 min (Figure 5).

### 2.5. Sample Washing of Silk Samples B.S.1-3 and R.S.1-3

Only the samples of the third test (B.S.1-3 and R.S.1-3) were washed by immersion in a 500 mL tap water bath (maintained at 35 °C) to which 4 mL of a liquid detergent was added, as the detergent: water ratio indicated on the packaging is 40 mL:5000 mL, with constant stirring. After 30 min, the samples were rinsed under running water (3 L) and air-dried at room temperature. Then, a second washing cycle in the same conditions and a third in which the temperature was maintained at 35 °C for 30 min and then increased to 65 °C for another 30 min were carried out. The temperature of the last wash was increased to 65 °C, although silk garments are generally washed at lower temperatures in order to evaluate the effect of additives even at “extreme” temperatures. After each washing cycle, the sample was air-dried at room temperature.

We obtained 3 series of 4 samples for each color labeled as described in Table 6.

### 2.6. The Characterization of Tested Samples by Colorimetric Analyses

Colorimetric analyses were performed on all samples using the ARW-45/0° Handheld Colorimeter (ARW Misure, via Monte Pasubio,137,36010 Zane VI, Italy) to evaluate the color changes of the samples differently dyed before and after the various wash cycles. The ARW-45/0° Handheld Colorimeter is a portable instrument that is usable on plastic, textiles, clothing, metals, paper, construction, paints and varnishes, packaging, and food in general.

**Figure 6:**
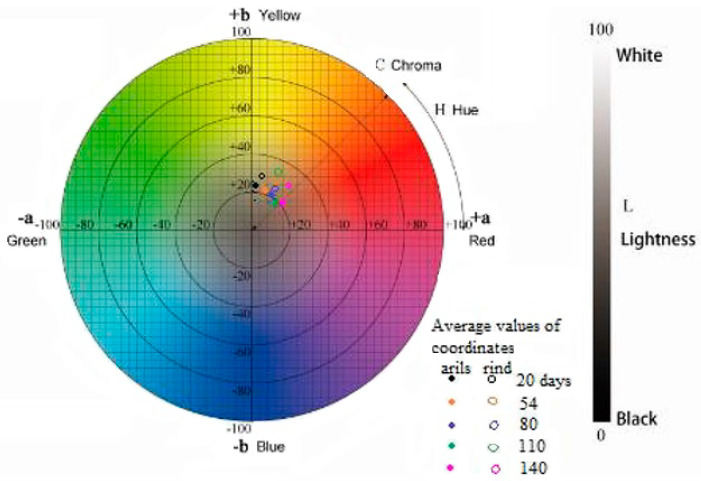
CIELab and CIELCh coordinates in the color space [19].

It is possible to measure color differences by estimating ΔE*, ΔL*, Δa* I Δb* according to formulas for CIE L*a*b* and CIE L*C*h* systems (Figure 6):

ΔE* = [(ΔL*)^2^ + (Δa*)^2^ + (Δb*)^2^]^1/2^

Δc* = [a_1_^*2^ + b_1_^*2^]^1/2^ − [a_2_^*2^ + b_2_^*2^]^1/2^

Δh* = [(ΔE*)^2^ − (ΔL*)^2^ − (C_ab_*)^2^]^1/2^

ΔE*, in this case, stands for the difference between the color of the sample before washing (standard color) and the color of the sample after washing. ΔL* represents a lightness difference between the two colors. Δa* stands for a redness or greenness difference between the two colors, and Δb* stands for a blueness–yellowness difference between the two colors.

The lower the ΔE* value, the closer the sample is to the standard. An ΔE* value of 0 means that the two colors are identical. The greater the ΔE* value, the greater the difference between the two colors, which, in our case, means that a greater part of the color was lost during washing.

Δc* is the difference in chroma between the color of the sample before washing (standard color) and the color of the sample after washing (+ = brighter, − = duller).

Δh* is the difference in hue between the color of the sample before washing (standard color) and the color of the sample after washing (+ = redder − = greener).

## 3. Results

### 3.1. Visual Evaluation of the Results of the Preliminary Tests

The entire procedure was documented photographically because some differences could be perceived visually.

**Figure 7:**
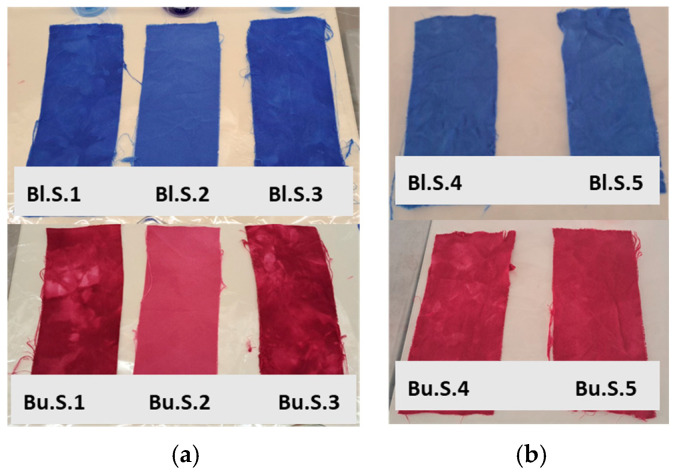
Comparison of the silk samples obtained after the first (**a**) and the second (**b**) tests.

The samples after the dyeing cycle are, in fact, visually different. The samples of the first group, to which nothing other than Acetic acid, Sodium sulfate, and Sodium acetate were added (Bl.S.1 and Bu.S.1), are darker and less homogeneous dyed than the ones where Albegal SET^®^ was added (Bl.S.2 and Bu.S.2). This result is not surprising, as the reason for adding Albegal SET^®^ is precisely to promote excellent dye distribution. In addition, these inhomogeneous samples show undyed areas (especially in the burgundy samples), which are probably due to the twisting of the textile during the dyeing cycle. However, the dyeing is also not homogeneous in the samples to which mucilage was added (Bl.S.3 and Bu.S.3) (Figure 7a).

These two observations prompted us to carry out the second test, i.e., the concentration of mucilage added to samples Bl.S.4 and Bu.S.4 was doubled, and the volume of water added to sample Bl.S.5 and Bu.S.5 was slightly increased so as not to distort the samples (Figure 7b).

The comparison of all samples shows that the best results were obtained when using mucilage with the highest liquor content (Bl.S.5 and Bu.S.5), while the concentration of the gel does not seem to play a role. Furthermore, when comparing the two dyes, the best results were obtained with LANASET^®^ BLUE 2RA.

**Figure 8:**
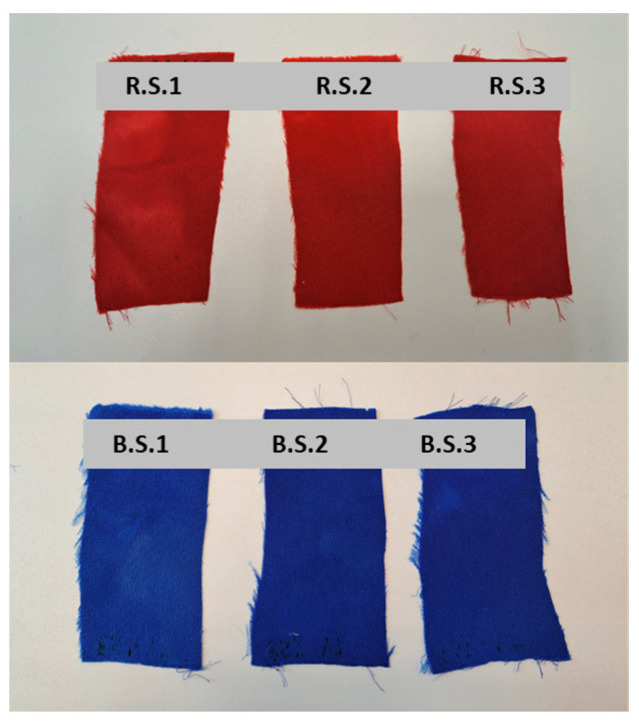
Comparison of the silk samples obtained after the dyeing cycles.

Also, the samples of the third group (dyed with LANASET^®^ Red 2GA-01 and LANASET^®^ and increased liquor content) are visually different after the dyeing cycle. The samples to which nothing other than acetic acid, sodium sulfate, and sodium acetate were added (R.S.1 and B.S.1) are the least homogeneous dyed (Figure 8), but from the comparison of the two other samples (R.S.2-R.S.3 and B.S.2-B.S.3), we can conclude that prickly pear mucilage has a very similar effect to Albegal SET^®^, although both samples are clearly more homogeneous.

Given this fact, we decided to mark an area on each sample to be checked with the colorimeter before and after each wash. In this way, we can be sure that any differences detected by the colorimeter are due to the washing and not to the different color intensities on the sample.

**Figure 9:**
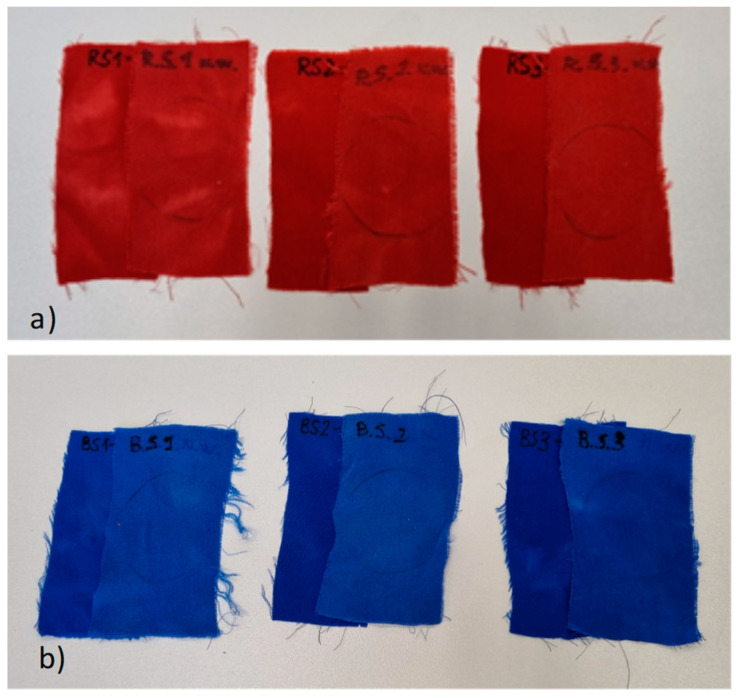
Visual color comparison between the silk samples before washing (**left**) and the samples after the washing cycles (**right**) for the red (**a**) and blue (**b**) samples.

The samples were also photographed after washing. The red color seems to be more stable than the blue one in all three cases. In both series, the samples treated with cactus mucilage (R.S.3 and B.S.3) appear to be the least discolored (Figure 9a,b).

**Figure 10:**
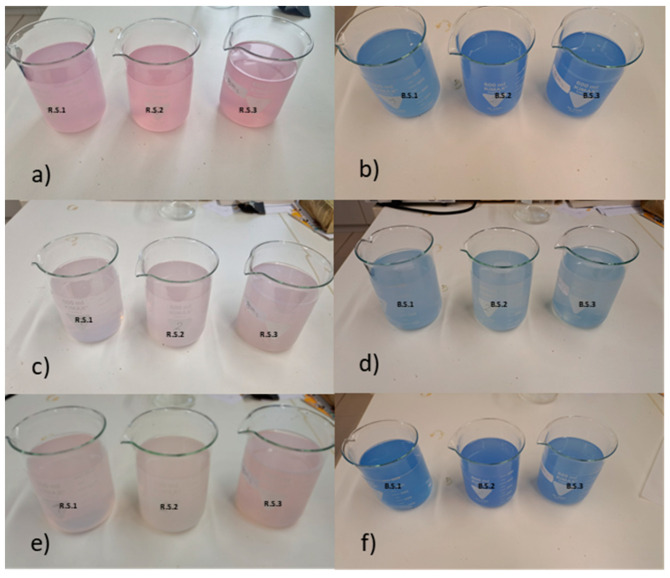
The wash waters of (**a**) the red-dyed silk samples and (**b**) the blue-dyed silk samples after the first wash, (**c**) the red-dyed silk samples and (**d**) the blue-dyed silk samples after the other washes, (**e**) the red-dyed silk samples and (**f**) the blue-dyed silk samples after the third wash.

After the first wash, the water bath became noticeably colored, which means that the dyed fabrics gave off color in the warm water. Figure 10a,b shows that samples R.S.1 and B.S.1 lost less color after the first wash than the other two. The pH value of all water baths was 7. After the second wash, this difference is no longer detectable for both dyes (Figure 10c,d). After the third wash, the water bath was visibly colored again due to the highest temperature (Figure 10e,f). For the blue samples, B.S.3 appears to have lost less color than the others (Figure 10f).

### 3.2. Results Obtained of the Colorimetric Analysis for Washed Samples

The results of the colorimetric analysis are given in Table 7, where the ΔE* values are reported as the measurement of the color difference between every sample and the standard, that is, the sample before washing.

The Δc* values are all negative with one exception, indicating lower color intensity in all samples, which is an obvious result given the loss of color after each wash.

On the contrary, the Δh* values are all positive, indicating that all samples in the L*C*h* system fall counterclockwise to the standards, i.e., that all samples are less red than the standards, which is consistent with the negative values for Δa* (for almost all samples) indicating the difference on the red/green axis (+ = redder − = greener).

**Figure 11:**
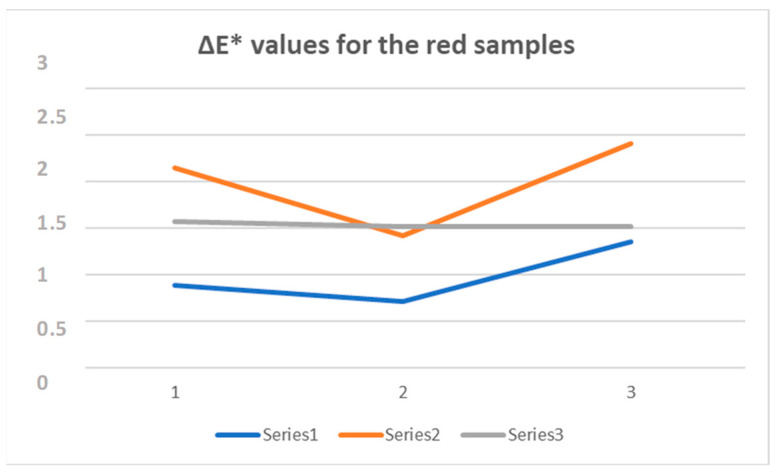
ΔE* graphic for the red series: Series1—R.S.1, Series2—R.S.2, Series3—R.S.3.

**Figure 12:**
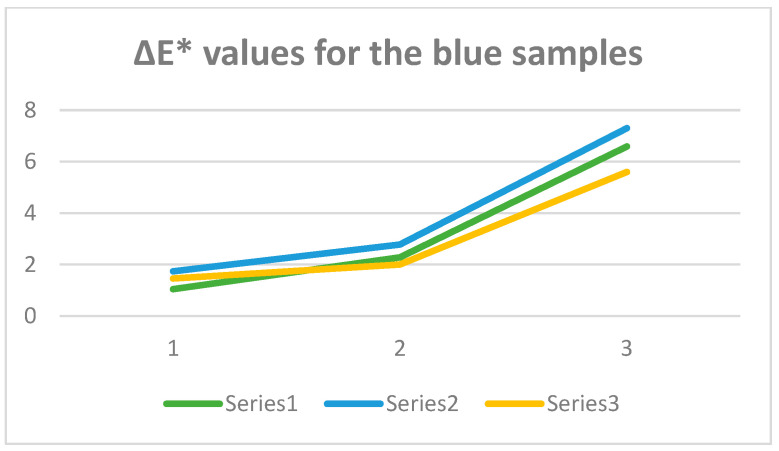
ΔE* graphic for the blue series: Series1—B.S.1, Series2—B.S.2, Series3—B.S.3.

The comparison of the ΔE* calculated for the series of samples is shown in Figure 11 and Figure 12 for red and blue samples, respectively.

Comparing the ΔE* measurements for the red silk samples, it can be seen that after washing at 35 °C, the most stable sample is R.S.1, while after washing at 65 °C, the most stable sample is R.S.3. It can also be seen that in the case of R.S.3, the color loss only occurred during the first wash, after which the ΔE* values were nearly constant as is evident from the graph in Figure 10, where for the third series, the curve obtained is almost a straight line.

Comparing the ΔE* measurements for the blue silk samples, it is evident that all three series show a similar trend; as evident from the graph in Figure 11, the color loss increases after every single wash treatment. In this case, it should be underlined that after the second and third washing, the best result is that of the B.S.3 series, i.e., the sample to which the mucilage has been added.

## 4. Discussion and Conclusions

**Figure 13:**
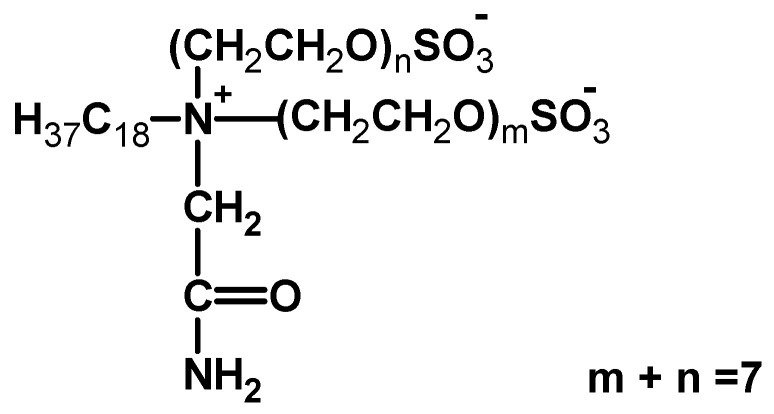
Amphoteric leveling agent for reactive dyeing.

The results obtained show that prickly pear mucilage could replace the commercially available chemical product Albegal SET^®^. The samples dyed with Albegal SET^®^ or gel are indeed both very homogeneously colored (when the liquor ratio is increased) and show similar wash fastness. In general, the use of surfactants during the dyeing cycle has the purpose of reducing the surface tension of the fibers. Albegal is an amphoteric surfactant (Figure 13) [20] that, at acidic pH values (such as the one we used), becomes cationic and can therefore bind to negatively charged acid dyes. However, these bonds are slowly broken thanks to the energy provided by the heating in the dyeing cycles. In this way, the dye is released slowly and absorbed more uniformly by the polymer system of the fiber. Without the surfactant, however, the dye is bound directly to the textile fiber, but in some areas, only on the surface [21].

Prickly pear mucilage is a polysaccharide containing protein parts (see Figure 4) that are thought to be responsible for their surface-active properties [22]. In this case, probably due to the hydrophobic attraction between dye and mucilage micelles, unstable complexes are formed that slowly release dye ions that are adsorbed more slowly and evenly to the surface of the silk fiber, and this is the reason why prickly pear mucilage delivers a similar result to Albegal SET^®^.

The advantage of using mucilage is, of course, its nature, i.e., a biopolymer obtained from industrial waste that is easily accessible, safe, and sustainable, but on the other hand, mucilage seems to require a greater amount of water, which is still a shortcoming from the point of view of environmental sustainability. In conclusion, our study shows the potential of prickly pear mucilage as an additive for dyeing silk and that it would be appropriate to investigate further in this field in order to find the best conditions for its use during the dyeing cycle.

It is recognized that the complete reduction of the environmental impact of textile dyeing would require the use of natural dyes and additives. However, as natural dyes often do not meet market requirements, even a partial reduction of chemical agents is a positive step.

## Figures and Tables

**Table 1:** Weights in grams of the silk samples for the first test.

Sample	m (g)
Bl.S.1 ^1^	2.20
Bl.S.2	2.16
Bl.S.3	2.25
Bu.S.1 ^2^	2.21
Bu.S.2	2.21
Bu.S.3	2.20

^1^ Bl.S. means blue and silk, ^2^ Bu.S. means burgundy and silk.

**Table 2:** Weights in grams of the silk samples for the second test.

Sample	m (g)
Bl.S.4 ^1^	2.15
Bl.S.5	2.00
Bu.S.4 ^2^	2.20
Bu.S.5	2.20

^1^ Bl.S. means blue and silk, ^2^ Bu.S. means burgundy and silk.

**Table 3:** Weights in grams of the silk samples for the third test.

Sample	m (g)
B.S.1 ^1^	3.02
B.S.2	3.05
B.S.3	3.08
R.S.1 ^2^	3.06
R.S.2	3.01
R.S.3	3.05

^1^ B.S. means blue and silk, ^2^ R.S. means red and silk.

**Table 4:** Labeling and meaning of samples for first and second tests.

Sample	Additive ^1^	Liquor Ratio
Bl.S.1	-	1:40
Bl.S.2	Albegal SET^®^	1:40
Bl.S.3	Mucilage (*Opuntia ficus indica*)	1:40
Bl.S.4	Mucilage (*Opuntia ficus indica*) ^2^	1:40
Bl.S.5	Mucilage (*Opuntia ficus indica*)	1:60
Bu.S.1	-	1:40
Bu.S.2	Albegal SET^®^	1:40
Bu.S.3	Mucilage (*Opuntia ficus indica*)	1:40
Bu.S.4	Mucilage (*Opuntia ficus indica*) ^2^	1:40
Bu.S.5	Mucilage (*Opuntia ficus indica*)	1:60

^1^ additive added in addition to acetic acid, sodium sulfate, and sodium acetate. ^2^ double concentration of mucilage.

**Table 5:** Labeling and meaning of the silk samples for the third test.

Sample	Additive ^1^	Liquor Ratio
B.S.1	-	1:100
B.S.2	Albegal SET^®^	1:100
B.S.3	Mucilage (*Opuntia ficus indica*)	1:100
R.S.1	-	1:100
R.S.2	Albegal SET^®^	1:100
R.S.3	Mucilage (*Opuntia ficus indica*)	1:100

^1^ additive added in addition to acetic acid, sodium sulfate, and sodium acetate.

**Table 6:** Labeling and meaning of the first group of silk samples.

Labeling	Meaning
B/R.S.i ^1^ NW	not washed
B/R.S.i 1W	1st wash for 30 min, T = 35 °C
B/R.S.i 2W	2nd wash for 30 min, T = 35 °C
B/R.S.i 3W	3rd washed for 30 min, T = 35 °C and 30 min, T = 65 °C

^1^ i = 1–3.

**Table 7:** ΔE* values for all the silk samples.

Sample	ΔL*	Δa*	Δb*	Δc*	Δh*	ΔE* ^1^
R.S.1 1W	−0.05	−0.44	−0.77	−0.76	0.47	0.89
R.S.1 2W	−0.69	0.01	0.19	0.10	0.16	0.71
R.S.1 3W	1.16	−0.43	−0.53	−0.63	0.26	1.35
R.S.2 1W	−0.93	−1.30	−1.43	−1.82	0.64	2.15
R.S.2 2W	0.05	−0.75	−1.20	−1.23	0.71	1.42
R.S.2 3W	1.93	−0.74	−1.23	−1.24	0.74	2.41
R.S.3 1W	−1.01	−1.03	−0.63	−1.20	0.05	1.57
R.S.3 2W	0.37	−0.50	−1.39	−1.11	0.98	1.52
R.S.3 3W	0.84	−0.74	−1.03	−1.15	0.54	1.52
B.S.1 1W	0.89	0.15	0.54	−0.52	0.21	1.05
B.S.1 2W	1.91	−0.91	0.85	−0.93	0.82	2.28
B.S.1 3W	5.88	−1.93	2.28	−2.43	1.74	6.59
B.S.2 1W	0.70	−0.46	1.53	−1.56	0.33	1.74
B.S.2 2W	1.73	−1.09	1.88	−1.96	0.94	2.78
B.S.2 3W	5.89	−2.06	3.79	−3.92	1.80	7.30
B.S.3 1W	1.04	−0.79	0.64	−0.75	0.69	1.46
B.S.3 2W	1.32	−1.25	0.85	−1.01	1.12	2.00
B.S.3 3W	4.35	−2.88	2.04	−2.37	2.62	5.60

^1^ The colorimeter used has a standard deviation within ΔE*ab 0.03. Average of 30 measurements of standard white plate.

## Data Availability

The original contributions presented in the study are included in the article, further inquiries can be directed to the corresponding author.
